# Roles of zinc and metallothionein-3 in oxidative stress-induced lysosomal dysfunction, cell death, and autophagy in neurons and astrocytes

**DOI:** 10.1186/1756-6606-3-30

**Published:** 2010-10-26

**Authors:** Sook-Jeong Lee, Jae-Young Koh

**Affiliations:** 1 Neural Injury Research Center, Department of Neurology, Asan Institute for Life Science, University of Ulsan, College of Medicine, Seoul 138-736, Korea; 2 Neural Injury Research Center, Asan Institute for Life Science, University of Ulsan, College of Medicine, Seoul 138-736, Korea

## Abstract

Zinc dyshomeostasis has been recognized as an important mechanism for cell death in acute brain injury. An increase in the level of free or histochemically reactive zinc in astrocytes and neurons is considered one of the major causes of death of these cells in ischemia and trauma. Although zinc dyshomeostasis can lead to cell death via diverse routes, the major pathway appears to involve oxidative stress.

Recently, we found that a rise of zinc in autophagic vacuoles, including autolysosomes, is a prerequisite for lysosomal membrane permeabilization and cell death in cultured brain cells exposed to oxidative stress conditions. The source of zinc in this process is likely redox-sensitive zinc-binding proteins such as metallothioneins, which release zinc under oxidative conditions. Of the metallothioneins, metallothionein-3 is especially enriched in the central nervous system, but its physiologic role in this tissue is not well established. Like other metallothioneins, metallothionein-3 may function as metal detoxicant, but is also known to inhibit neurite outgrowth and, sometimes, promote neuronal death, likely by serving as a source of toxic zinc release. In addition, metallothionein-3 regulates lysosomal functions. In the absence of metallothionein-3, there are changes in lysosome-associated membrane protein-1 and -2, and reductions in certain lysosomal enzymes that result in decreased autophagic flux. This may have dual effects on cell survival. In acute oxidative injury, zinc dyshomeostasis and lysosomal membrane permeabilization are diminished in metallothionein-3 null cells, resulting in less cell death. But over the longer term, diminished lysosomal function may lead to the accumulation of abnormal proteins and cause cytotoxicity.

The roles of zinc and metallothionein-3 in autophagy and/or lysosomal function have just begun to be investigated. In light of evidence that autophagy and lysosomes may play significant roles in the pathogenesis of various neurological diseases, further insight into the contribution of zinc dynamics and metallothionein-3 function may help provide ways to effectively regulate these processes in brain cells.

## Introduction

Cells have two major protein degradation pathways: the ubiquitin-proteasome system (UPS), which mainly acts to clear and recycle short-lived proteins [1], and macroautophagy or autophagy, in which lysosomal degradation is the final event [2]. This latter pathway degrades waste proteins and organelles, recycling damaged organelles and large proteins that cannot be processed via the UPS. The autophagic pathway usually operates at low levels under normal conditions, but is rapidly upregulated under stress conditions, such as starvation, hormonal imbalances, and oxidative stress [2-4]. Whereas autophagic degradation releases free amino acids and fatty acids that serve to meet the energy demands of cells in starvation [5], it also removes potentially detrimental abnormal organelles and misfolded proteins [6].

During the last decade, abnormalities in autophagy have been suggested to play roles in the pathogenesis of cancer and neurodegenerative disease, among other disorders [7-15]. For instance, a reduction in autophagy is observed in various cancer cells [16-18], and internal or external activators of autophagy, such as Beclin-1 (BECN1), transforming growth factor-β (TGF-β), and rapamycin, have been shown to effectively reduce tumor mass in human hepatocellular carcinoma cells and xenografted breast cancer cell lines [19-21]. There is also evidence for reduced or blocked autophagy in various neurodegenerative conditions, including Alzheimer's disease, Parkinson's disease, Niemann-Pick type C disease, and Huntington's disease [22-26]. Consistent with this, downregulation of autophagy-activating genes in the brain results in severe neurodegeneration [23,27,28]. Given the potential clinical importance of autophagy, there has been rapidly increasing interest in investigating this process in various disease models.

Recently, we reported that zinc and metallothionein 3 (MT3) have modulatory effects on autophagic vacuole (AV) formation and lysosomal changes in cultured brain cells [29-31]. Zinc serves many essential functions in the body under normal conditions; it is enriched in all cells, and is absolutely required for cellular development and survival [32,33]. Accordingly, a severe zinc deficiency causes developmental anomalies in humans and animals [34-36]. On the other hand, increased free zinc levels in a cell can be highly cytotoxic. The toxic role of endogenous zinc has been extensively studied, especially in the context of acute brain injury [37-41], where zinc has been shown to be capable of causing cell death through diverse mechanisms. For instance, high levels of intracellular free zinc can activate protein kinase C (PKC), nicotinamide adenine dinucleotide phosphate (NADPH) oxidases [42], p38 mitogen-activated protein kinase (MAPK) [43-45], poly ADP-ribose polymerase (PARP) [46,47], p75^NTR^-associated death executor (p75^NTR^/NADE), and apoptosis-inducing factor (AIF) [48-50].

Because cells are vulnerable to drastic changes in intracellular free zinc, they are equipped with a number of proteins that function to regulate zinc levels. For instance, zinc transporters (ZnTs) and Zrt- and Irt-like proteins (ZIPs) function to transfer zinc across membranes [51,52]. In addition, cysteine-rich metallothioneins may function as zinc buffers inside cells. Of the metallothioneins (MTs), MT3 is especially enriched in the brain [53,54]. Some MT3 zinc-binding sites are redox modulated, allowing MT3 to accept and release zinc in response to changes in oxidative status [53-57]. Because MT3 can induce or reduce zinc toxicity depending on context, it may increase or decrease brain injury, depending on the particular state of MT3. For example, if apo-forms are predominant, MT3 may accept zinc, acting as a buffer against rising intracellular zinc levels. In contrast, if zinc-binding cysteine residues of MT3 are oxidized, MT3 may release zinc and cause more cell death.

However, our recent findings suggest that MT3 may have more complex effects on cell biology than simply functioning as a zinc buffer. For instance, astrocytes from MT3-null mice show altered activity of lysosomes--the endpoint in the autophagy pathway [31]. Here, we review the possible roles of zinc and MT3 in autophagy activation and lysosomal changes under oxidative stress conditions.

### Increases in Zinc under Oxidative Stress Conditions: Role in Neuronal and Glial Cell Death

The central nervous system contains high levels of zinc, which is present at about 70-80 ppm in gray matter [58]. Whereas the majority of brain zinc is tightly bound to proteins, about 10-20% is localized to certain glutamatergic vesicles in a relatively free state (chelatable zinc) [59-61]. This synaptic zinc may be released upon neuronal activation, and is involved in signal transmission/transduction across synapses [35,62-65]. However, in acute brain injury, the rise of intracellular free zinc levels contributes to neuronal and astrocytic cell death [37,66-70]. For example, zinc-induced neurotoxicity is observed following acute brain injury, such as trauma, seizures, and ischemia [35,37,38,71-75]. Whereas synaptic zinc may trigger toxic cascades in areas such as the hippocampal CA3 region, where synaptic zinc is especially enriched in mossy fiber terminals [76,77], intracellular zinc release may play a larger role in most other brain regions [77-79].

Calcium-overload excitotoxicity is still considered to be the major mechanism of neuronal death in acute brain injuries, including focal ischemia [80-82]. However, calcium excitotoxicity alone may not be a sufficient to produce infarcts, in which astrocytes and oligodendrocytes, which are much less vulnerable to glutamate [83-85], are also severely damaged. Hence, factors that contribute to non-neuronal cell death need to be identified. In our previous study, we found that the infarct core exhibits markedly increased levels of labile zinc in all cellular elements [86,87], raising the possibility that zinc toxicity may contribute to infarct formation. In fact, oxidative stress, which usually accompanies focal ischemia, induces increases in labile zinc in astrocytes as well as neurons [37,88-90].

So, which toxic mechanisms does zinc trigger inside cells? Studies over the last decade have suggested a number of different mechanisms that may mediate zinc neurotoxicity. Activation of PKC, NADPH oxidases, extracellular signal regulated kinase 1/2 (ERK-1/2), and PARP by zinc has been shown to cause mainly oxidative neuronal necrosis [35,42]. In addition, caspase-mediated apoptosis is induced by the activation of the p75^NTR^/NADE pathway and by AIF released from mitochondria in zinc-exposed neurons [48-50,91].

### Lysosomal Membrane Permeabilization and Zinc

**Figure 1:**
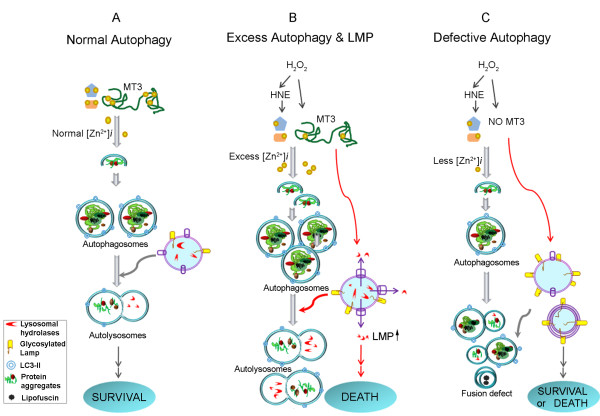
**Summary diagram depicting zinc and MT3 effects on autophagy and lysosomes**. A. Under physiological conditions, normal signaling involving zinc release from MT3 contributes to the normal progression of autophagy, resulting in the degradation of abnormal protein aggregates and waste organelles. This pathway may be beneficial for cell survival. B. Under injurious oxidative stress conditions (e.g., H_2_O_2 _or HNE treatment), the events described in (A) are exaggerated. Hence, much more intense zinc release from MT3 is induced, and excess autophagy is activated. Excess autophagy and excess zinc accumulation in lysosomes ultimately leads to LMP and cell death. C. Downregulation of MT3 decreases zinc release from MT3, inhibiting lysosomal functions and reducing fusion between autophagosomes and lysosomes, resulting in a reduced autophagy flux. Under conditions of acute injury, this results in a reduction in both LMP and cell death, but it can be detrimental to cell survival under conditions of chronic stress by reducing autophagic degradation of abnormal proteins.

In addition to the above-mentioned mechanisms for zinc toxicity, we have recently presented evidence that lysosomal changes may underlie zinc-induced cell death (Figure 1) [29]. The lysosome is an acidic cytosolic vesicle that contains numerous (>80) acidic hydrolases--glycosidases, phosphatases, proteases, nucleases, peptidases, sulphatases and lipases--that collectively are capable of degrading all cellular components. As such, the lysosome serves as the main degradative factory in cells, receiving cargoes from phagosomes, endosomes, and autophagosomes. Because lysosomal acidic hydrolases are so potent, their release in combination with cytosolic acidification can cause cell death through severe breakdown of cellular components as well as activation of cell death inducers, such as BID. This process is termed lysosomal membrane permeabilization (LMP) [92].

LMP has been shown to occur in cell death caused by oxidative stress, calcium overload, p53 activation, and exposure to lysosomotrophic toxins such as sphingosine [92,93]. Moreover, several cancer chemotherapeutic agents have been shown to induce lysosomal changes, including LMP, in diverse cancer cell types [2]. In the brain, epileptic injury and ischemic injury may cause LMP in certain neurons, inducing their death [5,8,29], and lysosomal enzyme inhibitors may be neuroprotective against ischemic insults [94-96].

Recently, we presented evidence that LMP is a key contributor to oxidative and zinc-induced hippocampal neuronal death [29]. The salient features of this mechanism are as follows: Under normal conditions, free zinc levels in lysosomes are low. Following exposure to H_2_O_2 _or toxic levels of zinc, the level of zinc in lysosomes rises rapidly and significantly. Next, a substantial fraction of zinc-laden lysosomes undergo membrane disintegration, releasing enzymes such as cathepsins. Finally, hippocampal neuronal death occurs in a zinc- and cathepsin-dependent manner. These results indicate that zinc-overload in lysosomes and lysosomal disruption are key events in oxidative neuronal death (Figure 1). Interestingly, lysosomes also accumulate 4-hydroxy-2-nonenal (HNE) adducts in a zinc-dependent manner, and HNE per se causes LMP, suggesting that HNE may be one of mediators of lysosomal derangement in oxidative and/or zinc-mediated neuronal death (Figure 1). Further studies will be needed to firmly establish the relationship between known signaling events in zinc toxicity and LMP.

The role of various organelles in cell death has been extensively studied in recent years. Initially, the focus was on mitochondria, since this organelle is the main site of reactive oxygen species (ROS) generation and because it also contains and/or releases several apoptosis-regulating molecules, including B-cell lymphoma 2 (Bcl-2), Bcl-2-associated X protein (Bax), AIF, and cytochrome c [15]. More recently, the endoplasmic reticulum (ER) has received increasing research attention. ER stress plays an integral role in the unfolded protein-induced alarm system that activates multiple signaling pathways, including MAPK, c-Jun N-terminal kinase (JNK), p38 MAPK, and nuclear factor-κB (NF-κB) pathways [97-101]. Similarly, a recent surge in interest in autophagy has brought the lysosome to the fore as another organelle with a major role to play in cell death mechanisms. In this context, the possibility that cellular free zinc may function as a link between oxidative stress and LMP is particularly intriguing.

Then what is the mechanism underlying lysosomal zinc accumulation? One possible answer is that the accumulation of zinc in lysosomes may be an exaggerated version of a normal physiologic event, involving the transport of zinc from the cytosol via certain metal transporters or ionophores. In this case, zinc may serve as an activator of lysosomal functions. Alternatively, zinc accumulation may merely be a result of lysosomal activation, reflecting zinc release from various zinc-binding proteins inside lysosomes. Although it is unclear which is the case, the demonstration that the cell-permeant zinc chelator TPEN (tetrakis[2-pyridylmethyl]ethylenediamine) not only blocks the rise in free zinc levels in lysosomes but also inhibits LMP tends to favor the former possibility. The precise inter-organelle zinc dynamics within cells warrant further investigation.

### Autophagy and Zinc

The finding that zinc dyshomeostasis is closely connected to lysosomes, the effector organelle for autophagy, prompted us to investigate the possible role of zinc in the entire autophagic cascade. Autophagy means "self-eating" in Greek [6,102]. It is evolutionally conserved in all eukaryotes and serves the essential self-digestive function of degrading large proteins and organelles [3,103].

Of the three known types of autophagy--macroautophagy, microautophagy, and chaperone-mediated autophagy [6,104,105]--macroautophagy (or simply autophagy) is the best characterized. Recent advances in the molecular biology of autophagy have led to the identification of a number of proteins required for this process, including the autophagy-related homologs, BECN1 and ATG5. Microtubule-associated membrane protein-II (LC3-II), another autophagy-related protein, is inserted into the outer membrane of autophagosomes and has been used as a marker for autophagic activation [106,107]. LC3-II is quite stable and hence easy to detect. Transfection with GFP- or RFP-LC3 has been widely used to monitor the autophagic process in living cells.

Recently, we used this approach in cultured astrocytes, which are easier to transfect than primary neurons. In astrocytes, inducers of oxidative stress such as H_2_O_2 _or FeCl_2 _activate autophagy, as evidenced by increased LC3-II levels and autophagosome formation (Figure 1). Interestingly, zinc accumulation occurs in autophagosomes as it does in lysosomes [30]. Importantly, TPEN blocks the activation of autophagy by oxidative stress, suggesting that zinc accumulation has already started at the level of autophagosomes and plays a role in autophagy progression. It is not yet known whether specific zinc transporters are responsible for the zinc accumulation. Moreover, it is possible that TPEN effect is not due to chelation of zinc inside autophagosomes, but may instead reflect effects on upstream elements in the signaling cascade, such as inhibition of phosphatidylinositol 3-kinase type III (PI3K type III) activation. In either case, these data represents the first demonstration that cellular zinc may play a role in activation of the autophagic process. Whether autophagy thus activated is functional (i.e., increases autophagic flux) will require additional study; however, the fact that mutant huntingtin protein (mHttp) aggregation is reduced under these conditions suggests that this may be the case.

One interesting question is whether the role of zinc in autophagy and LMP is limited to brain cells or is more generally applicable to other cell types. The fact that tamoxifen-induced autophagic cell death in MCF-7 breast cancer cells [14] exhibits similar features, such as zinc accumulation in AVs and LMP [29,30], suggests that zinc may play a role in autophagy and autophagic cell death in general. If confirmed, which will require addition testing in other cell types and diverse models, modulation of zinc levels may prove to be an effective therapeutic intervention under conditions in which abnormalities in autophagy are contributing factors, such as cancer and neurodegenerative disorders.

### MT3: the Source for Zinc in Neurons and Astrocytes

Human genome analyses have revealed that more than a thousand proteins may contain zinc-binding motifs [108]. However, most of these proteins bind zinc tightly, and hence may not normally contribute to fast zinc dynamics in cells. In contrast, some proteins, such as MTs, contain zinc-binding sites that are highly sensitive to redox states [25,109-114]. When cells are exposed to reducing conditions or when cellular free zinc levels rise, apo-forms of MTs (thioneins) may bind more zinc. Conversely, under conditions in which cells are exposed to oxidative stress and during signaling events involving ROS generation, MTs may serve as zinc-donors, raising free zinc levels (Figure 1) [114]. Consistent with this, diverse cells exhibit a rise in free zinc levels in response to stressful extracellular or intracellular stimuli, including hormones, cytokines, metals, inflammation, oxidative agents, and other stresses [115-118]. This increase in free zinc may stimulate diverse cellular-response signals [109,110,119,120]

Four isoforms of MT, MT1-4, have been identified in mammals, all of which have seven metal binding domains [113,121-124]. MT1 and MT2 are ubiquitously expressed in all tissues. In contrast, MT3 and MT4 are expressed mainly (although not exclusively) in the central nervous system and squamous epithelia [125-128]. Hence, it is likely that, at least in the brain, MT3 may serve as a key source for dynamically exchangeable zinc in cells exposed to various stress stimuli.

MT3 was originally identified as a neuronal growth inhibitory factor that inhibited outgrowth of rat cortical neurons in the presence of Alzheimer's disease brain extracts [57]. This effect is not shared by MT1 or MT2, and is probably due to the unique presence of a TCPCP motif within the β-domain of MT3 [117,122,129-131]. The precise mechanism underlying the neurite outgrowth-inhibitory effect of MT3 remains poorly understood, but a number of studies have implicated MT3 in various neurological conditions. Altered MT3 expression has been also reported in amyotrophic lateral sclerosis (ALS) [54], Down syndrome [132], pontosubicular necrosis [133], Parkinson's disease, meningitis, and Creutzfeld-Jakob disease [134].

In addition, MT3 appears to exert both protective and injury-promoting effects in experimental models of brain injury. The neuroprotective effects of MT3, which are presumably due to its metal-chelating and antioxidative effects, are evident in epileptic brain injury [135], cortical cryolesions [136], a mutant superoxide dismutase 1 (SOD1[G93A]) mouse model of ALS [137], and peripheral nerve injury [138]. Several researchers have demonstrated the opposite phenomenon, showing for example that intracellular zinc released from MT3 may trigger neuronal death in vivo [77] and in vitro [31], indicating the injury-promoting effects of MT3 [54,56].

In adult brains, MT3 is predominantly expressed in neurons, but in developing brains it is also significantly expressed in astrocytes. We have demonstrated that the increase in intracellular free zinc induced by oxidative injury is significantly reduced in cultured MT3-null astrocytes compared with wild-type cells (Figure 1) [31]. Moreover, cell death is also attenuated in MT3-null cells. These results provide additional support for the idea that MT3 is the main source for elevations in toxic free zinc in acute brain injury. Interestingly, although astrocytes express substantial amounts of MT1 and MT2 [120], experiments employing small interfering RNAs suggest that these MTs do not participate as zinc donors.

### Regulation of Lysosomal Functions by MT3

MT3 may have complex biological functions that extend well beyond its role as a simple buffer for zinc, as exemplified by its initial identification as a neuronal growth inhibitory factor. Recently, we found that MT3 may regulate the levels of lysosomal proteins, thus regulating the function of lysosomes [31]. Specifically, the absence of MT3 in astrocytes results in changes in the levels of LMP and altered LMP glycosylation patterns (Figure 1). Such changes may inhibit docking of upstream vesicles, such as autophagosomes and endosomes, to lysosomes. In fact, the levels of autophagic markers such as LC3-II are markedly increased in astrocytes from MT3-null mice (Figure 1).

Another interesting change induced by the absence of MT3 is a reduction in certain hydrolase activities that implies a decrease in lysosomal protein degradative capability. The combination of reduced zinc levels and reduced lysosomal enzyme levels may act to attenuate LMP and cell death (Figure 1). Moreover, decreased lysosomal function should lead to diminished autophagic flux. Consistent with this, we found that cholesterol metabolism was altered and mHttp aggregates accumulated in the absence of MT3 [31].

How does MT3 regulate lysosomal functions? Although the available information provides little insight into this question, one report notes that disruption of c-Abl, a member of the Abelson family of cytoplasmic non-receptor tyrosine kinases, has effects on lysosomes in the A549 alveolar carcinoma cell line that are similar to those observed in brain cells lacking MT3[139]. This suggests that MT3 may be involved in the c-Abl signaling cascade in brain cells. Alternately, adequate free zinc levels may be required to maintain normal lysosomal function. Further studies may help determine the mechanism(s) underlying MT3 effects on lysosomes. Regardless of the mechanism, because lysosomal function is linked to autophagy and neurodegenerative disorders, MT3 may prove to be a suitable target for drugs designed to control lysosomal function. By reducing toxic zinc accumulation, LMP, and cell death, downregulation of MT3 function may be beneficial in acute brain injury (Figure 1). Conversely, in neurodegenerative conditions in which the accumulation of protein aggregates contributes to the pathology, upregulation of MT3, and the associated enhancement in lysosomal function and protein degradation, may be beneficial (Figure 1).

## Conclusions

Recent studies have revealed that free zinc levels change in various organelles in response to physiologic or pathological stimuli, and suggest important functional consequences of these zinc dynamics. One aspect of this larger picture--the possible roles of free zinc in autophagic and lysosomal functions--has been the focus of this review. Under oxidative stress conditions, free zinc levels in the cytosol and lysosomes of cultured neurons and astrocytes rise, ultimately resulting in LMP and cell death. While this change may contribute to the cell death that occurs after acute brain injury, the fact that free zinc levels rise in AVs (autophagosomes and autolysosomes) following various stimuli is also notable. Because reducing the levels of free zinc with TPEN blocks all these changes, the rise of free zinc in AVs may play a role in the progression of the autophagic cascade. In brain cells, the source of free zinc may be MT3. In support of a role for MT3 in lysosomal function, the absence of MT3 results in drastic changes in the levels of lysosomal proteins and results in reduced lysosomal degradative capacity. Further studies will be needed to elucidate the mechanism by which MT3 regulates lysosomal functions.

## Competing interests

The authors declare that they have no competing interests.

## Authors' contributions

All authors participated in developing and discussing the ideas, integrating the information, and writing the manuscript. All authors have read and approved the final manuscript.
